# An intelligent reinforcement learning enhanced improved LEACH protocol for prolonging wireless sensor network lifetime

**DOI:** 10.1038/s41598-026-65225-w

**Published:** 2026-08-24

**Authors:** Hamdy H. El-Sayed, Elham M. Abd-Elgaber, Shereen K. Refaay

**Affiliations:** Faculty of Computers and Artificial Intelligence, Sohag University, Sohag, 82511 Egypt

**Keywords:** WSN, Lifetime, Throughput, Energy, Overhead, LEACH, ILEACH, RL-LEACH, RL-ILEACH, Engineering, Mathematics and computing

## Abstract

This study suggests an intelligent clustering protocol in Wireless Sensor Networks, called RL-ILEACH, which incorporates Reinforcement Learning (RL) into the ILEACH (Improved Low-Energy Adaptive Clustering Hierarchy) framework for adaptive and energy-aware CH selection in order to overcome these drawbacks. An RL agent is used in the suggested RL-ILEACH protocol to learn the best CH selection strategies depending on current network conditions, such as residual energy, node density, and communication distance. RL-ILEACH reduces energy dissipation and improves load balancing during intra-cluster and inter-cluster communication phases by dynamically adjusting CH selection options. To assess RL-ILEACH’s performance against traditional procedures, such as LEACH, ILEACH, and other cutting-edge clustering techniques, extensive simulation tests are carried out. According to simulation studies, RL-ILEACH performs noticeably better than current protocols, resulting in reduced total energy consumption, a longer stability period, a greater packet delivery ratio, and a longer network lifetime. In particular, RL-ILEACH minimizes early node failures brought on by uneven energy depletion and sustains a larger number of active nodes throughout subsequent rounds. RL-ILEACH is a reliable and scalable solution for energy-efficient clustering in dynamic Wireless Sensor Network settings, as these enhancements demonstrate that the incorporation of Reinforcement Learning permits intelligent, adaptive decision-making. RL-ILEACH prolonged the network lifetime by $\:784\:\:$% compare to LEACH. RL-ILEACH outperformed ILEACH by increasing the network lifetime by * 130*%. Moreover, relative to NNMH-LEACH, RL-ILEACH achieved a further * 108*% imporvment in network lifetime.

## Introduction

Wireless Sensor Networks (WSNs) are an essential element In modern intelligent systems. Environmental monitoring, industrial automation, military surveillance, smart cities, and healthcare systems are just a few of the numerous applications for WSNs. In order to detect, analyze, and send data to a central Base Station (BS) or Sink, a WSN usually comprises of a large number of tiny sensor nodes spread out over a geographic region. WSNs have a basic problem with energy restriction despite their broad use. Sensor nodes are often battery-powered and situated in hard-to-reach places, making it impracticable to repair or recharge their batteries. In order to provide a long network lifetime and dependable data delivery, energy efficiency is essential in the design of effective WSN protocols^1^.

Clustering-based routing protocols, which group nodes into clusters, have been created to reduce energy consumption. A distinct node known as the Cluster Head (CH) is in control each cluster. The CH gathers, aggregates, and sends data from member nodes to the BS. The Low Energy Adaptive Clustering Hierarchy (LEACH) is a particularly creative clustering approach. LEACH uses a probabilistic approach for CH selection and periodic cluster reformation to balance energy consumption among nodes. However, the random selection of CHs in LEACH might cause unequal energy consumption, resulting in the premature death of some nodes and poor network performance. To address these issues, various better versions have been developed, including better LEACH (ILEACH), which refines cluster formation and CH rotation depending on node energy and distance^2^. ILEACH improves energy efficiency compared to LEACH, but it still has limits due to static decision-making and a lack of adaptation to changing network circumstances.

Adaptive and intelligent CH selection procedures are required because to the dynamic nature of WSNs, which includes varied node capabilities, shifting network architecture, and varying node energy levels. Conventional protocols rely on pre-defined criteria or equations that are unable to respond to network changes in real time. Low-energy nodes might therefore still be chosen as CHs, which would shorten the network’s lifetime and stability and cause early node mortality. Recent developments in artificial intelligence (AI), namely RL, have created new avenues for the creation of flexible and self-learning network protocols. Through trial-and-error interactions with their surroundings, nodes can learn the optimum course of action to optimize cumulative rewards (such as energy efficiency or network lifetime). The system may learn dynamically when RL is applied to the CH selection process^3^.

Using RL in the CH selection process allows the system to dynamically learn which nodes are most suited to serve as CHs under current network conditions. Although ILEACH improves energy efficiency over the conventional LEACH technique, it nevertheless suffers from several issues:

- Randomness in CH selection may result in nodes with low residual energy or poor placement.
- Static thresholds cannot adjust to changing network circumstances or topology.
- Energy imbalance: Some nodes consume energy quickly, limiting total lifetime.
- Inadequate learning mechanisms: Decisions rely on static formulae rather than experience-based learning^4–6^.

As a result, an intelligent and adaptable CH selection system is required, capable of dynamically optimizing the clustering process in response to network conditions, energy levels, and topological changes.

As a result, an intelligent and adaptable CH selection system is required, capable of dynamically optimizing the clustering process in response to network conditions, energy levels, and topological changes. Integrating Reinforcement Learning with ILEACH is a possible option for overcoming these restrictions.

### Contributions of this work

The main contributions of this paper are summarized as follows:

1. A novel Reinforcement Learning-enhanced ILEACH protocol (RL-ILEACH) is proposed for WSNs, where the conventional heuristic-based CH selection mechanism of ILEACH is replaced with an adaptive Q-learning decision model capable of learning optimal CH selection policies from network interactions.
2. An intelligent CH selection framework is developed by modeling the clustering process as a reinforcement learning problem. The proposed agent makes CH selection decisions based on multiple network-state parameters, including residual energy, node degree, distance to the BS, and previous CH participation, enabling dynamic adaptation to changing network conditions.
3. A lightweight reward function is designed to jointly optimize residual energy, transmission cost, and successful packet delivery. The reward formulation balances energy efficiency with communication reliability while maintaining a low computational overhead suitable for resource-constrained sensor nodes.
4. A complete RL-ILEACH routing framework is presented, including the state space, action space, reward model, Q-learning update mechanism, and the overall clustering algorithm. The proposed design preserves the simplicity of ILEACH while introducing autonomous learning capabilities without requiring deep neural networks or centralized computation.
5. A comprehensive performance evaluation is conducted using MATLAB simulations under different network densities (200 and 500 sensor nodes). RL-ILEACH is compared with LEACH, ILEACH, RL-LEACH, and NNMH-LEACH using multiple performance metrics, including network lifetime, alive/dead nodes, energy consumption, throughput, communication overhead, and end-to-end delay.
6. Simulation results demonstrate significant performance improvements over existing clustering protocols. RL-ILEACH substantially extends network lifetime, improves energy balancing, increases the stability period, achieves higher packet delivery ratio and throughput, and reduces overall energy consumption. Specifically, the proposed protocol extends network lifetime by approximately 789% compared with LEACH, 196% compared with ILEACH, and 100% compared with NNMH-LEACH, while maintaining a larger number of active nodes throughout the simulation.
7. The proposed protocol provides a practical trade-off between intelligence and computational complexity. Unlike Deep Reinforcement Learning (DRL) and Multi-Agent Reinforcement Learning (MARL) approaches, RL-ILEACH employs lightweight tabular Q-learning, making it suitable for practical deployment in energy-constrained and large-scale Wireless Sensor Networks.

The rest of the paper is in Sect.  Related work: reviews related works on LEACH variants and reinforcement learning applications in WSNs. Section  Proposed RL-ILEACH protocol: describes the proposed RL-based ILEACH model, including its algorithmic structure and system design. Section  Experimental results and discussion discusses the simulation setup, performance metrics, and experimental results. Section  Conclusion and future work: concludes the research and provides recommendations for future work. Section  6: The reference work.

## Related work

The related work summarizes relevant research on energy-efficient routing and clustering in WSNs, with an emphasis on the LEACH, ILEACH, and RL clustering algorithms.

The purpose of this work is to evaluate the advantages and disadvantages of existing methodologies, highlight research needs, and present the justification for developing an RL-enhanced ILEACH procedure for efficient CH selection.

### Clustering in wireless sensor networks

Clustering is an extremely successful approach for decreasing energy consumption in WSNs. Clustering-based routing splits the network into groups known as clusters, which are managed by a CH. The CH collects data from cluster members, aggregates it, and sends it to the BS.

This hierarchical structure reduces the quantity of direct transmissions to the BS while balancing energy usage between nodes. However, the effectiveness of clustering procedures is strongly reliant on how CHs are chosen and clusters are created.

Key objectives of clustering in WSNs include:

- Minimizing energy consumption during data transmission.
- Ensuring uniform CH distribution across the network.
- Balancing energy load among all nodes.
- Maximizing the overall network lifetime.

### The compared protocols

The Low-Energy Adaptive Clustering Hierarchy (LEACH) protocol, established by Heinzelman et al. (2000), was of the earliest and most widely used hierarchical routing protocols in WSNs. LEACH operates in two main phases:

1. During the setup phase, nodes form clusters and elect CHs using a probabilistic threshold.
2. Steady-State Phase: CHs send aggregated data to the BS.

LEACH distributes energy demand equally among nodes by randomly rotating CHs. Despite its simplicity and energy-saving benefits, LEACH has some drawbacks.

- The distribution of CHs may be unequal throughout the network.
- Nodes with little residual energy may nevertheless be selected as CHs.
- The assumption that all nodes have equal energy and communication range is unrealistic for heterogeneous WSNs. In order to fix LEACH’s shortcomings, several updated versions have been created, the most famous of which being Improved LEACH (ILEACH). ILEACH adds techniques for selecting CH based on residual energy, node distance from the BS, and node position. This means that nodes with more energy and optimum locations are more likely to become CHs.

Key features of ILEACH include:

- Energy-based CH selection: Prevents low-energy nodes from becoming CHs.
- Distance-based clustering: Reduces the consumption of energy during transmission.
- Adaptive cluster building: improves network longevity and stability.

Although ILEACH improves energy balance and longevity over LEACH, it still relies on established calculations and criteria. These static algorithms cannot adjust to real-time network changes or unexpected energy depletion, making ILEACH less efficient in dynamic contexts. RL is a type of machine learning in which an agent interacts with its environment to learn optimal behavior. When the agent receives feedback for its actions in the form of rewards or penalties, it can ultimately determine the optimal course of action for optimizing cumulative rewards. RL can be utilized for intelligent routing and grouping decisions in WSNs^7^. The agent (a node or controller) chooses which node should become the CH by modeling CH selection as a Markov Decision Process (MDP), taking into account variables like:

- Residual energy,
- Distance to the BS,
- Node density,
- Previous performance metrics.

Common RL algorithms applied in WSNs include:

- Q-Learning is an RL technique that finds the value of state-action pairings without the need for a model.
- Deep Q-Networks (DQN) enable complex decision-making by combining RL and deep learning.
- Multi-Agent Reinforcement Learning (MARL) uses cooperative learning to accomplish a shared objective, like energy efficiency.

RL’s flexibility makes it ideal for dynamic environments where network conditions are ever-changing. NN-MH-LEACH is a new version of the LEACH protocol that incorporates multi-hop (MH) communication based on neural networks (NNs) for energy-conscious data transfer and intelligent CH selection. While multi-hop routing lowers long-distance transmission costs, the neural network uses metrics like residual energy, node density, and distance to learn the best CH election options. When compared to traditional LEACH and its variations, simulation findings show that NN-MH-LEACH greatly increases network lifetime, stability period, and energy efficiency. For large-scale and energy-constrained WSN applications, the suggested protocol is hence ideal^8–10^.

### RL-based cluster head selection approaches

Several studies have successfully applied RL to the cluster head selection problem in WSNs:

- Zhang et al. (2019)^4^ presented a Q-learning-based CH selection technique that modifies according to distance and residual energy. The results showed improved network lifetime and energy efficiency as compared to LEACH.
- Younis et al. (2020)^5^: used RL to develop a clustering framework for heterogeneous WSNs. Communication overhead was reduced by 20% as a result of the system’s adaptive CH selection.
- Kumar and Singh (2021)^6^: Introduced a Deep Reinforcement Learning model for CH selection that achieved better adaptability to topology changes.
- Agarwal et al. (2022)^7^: Proposed a hybrid RL-LEACH algorithm integrating Q-learning with LEACH, improving stability period and throughput significantly.

These studies demonstrate that RL can effectively overcome the limitations of static and heuristic-based CH selection by enabling real-time learning and adaptation^11–14^.

### Recent trends in hybrid energy-efficient clustering algorithms for WSNs

Recent research has increasingly focused on hybrid intelligent clustering algorithms that combine optimization techniques, machine learning, bio-inspired algorithms, and security mechanisms to improve the energy efficiency and lifetime of WSNs. These approaches attempt to overcome the limitations of conventional LEACH-based routing by introducing adaptive decision-making and multi-objective optimization.

Lonkar and Karmore^15^ proposed BCEWN, a hybrid bio-inspired clustering framework that integrates energy-aware routing with bio-inspired optimization techniques to improve cluster formation and energy utilization. The proposed model demonstrated significant improvements in network lifetime and energy consumption compared with traditional clustering protocols. However, the routing decisions are primarily optimization-driven and do not incorporate experience-based learning capable of adapting to continuously changing network conditions.

In another study, Lonkar et al.^16^ introduced an optimal hybrid energy-saving CH selection algorithm based on multiple optimization objectives. Their empirical evaluation demonstrated improvements in CH distribution, residual energy utilization, and overall network lifetime across different deployment scenarios. Nevertheless, the CH selection process remains dependent on predefined optimization criteria and lacks an adaptive learning mechanism that can dynamically modify decisions according to changing network states.

Earlier, Lonkar and Karmore^17^ conducted a comprehensive statistical evaluation of several power-aware routing protocols for wireless networks. Their study compared the energy efficiency and communication performance of existing routing techniques and highlighted the importance of balancing routing overhead with energy consumption. Although the work provides valuable comparative insights, it does not introduce an adaptive routing methodology capable of learning from previous network behavior.

More recently, Lonkar and Karmore^18^ proposed EBBSMWN, an energy-aware blockchain-based bio-inspired security model for wireless networks. The framework enhances communication security while maintaining energy efficiency through the integration of blockchain technology and bio-inspired optimization. Although the proposed approach significantly strengthens network security, the additional blockchain operations introduce computational and communication overhead that may not be suitable for highly resource-constrained sensor nodes.

Furthermore, Lonkar et al.^19^ presented a comprehensive review of hybrid clustering and routing protocols addressing the hotspot problem in WSNs. Their survey concluded that hybrid intelligent algorithms consistently outperform conventional routing protocols in terms of energy balancing and network lifetime. However, it also identified several open research challenges, including adaptive CH selection, lightweight intelligent routing, computational complexity reduction, and scalable learning-based solutions for dynamic WSN environments.

Juwaied and Jackowska-Strumillo^20^ evaluated machine learning techniques, including K-Means and K-Nearest Neighbors (KNN) alongside Deterministic Energy-Efficient Clustering (DEC), to optimize WSN node selection and energy efficiency. Both variants, however, rely on distance-based, largely static ML techniques (supervised KNN and unsupervised K-Means) rather than an online, experience-driven decision process: cluster heads are recomputed periodically from fixed rules rather than learned adaptively from reward feedback. In contrast, RL-ILEACH embeds a lightweight tabular Q-learning agent directly into the cluster-head selection process of ILEACH, allowing the CH policy to continuously adapt to residual energy, node density, and distance as the network evolves, rather than being re-derived at fixed intervals. This distinction, learned feedback-driven adaptation versus periodic distance/centroid re-computation, is the primary conceptual advantage RL-ILEACH offers over the DEC-KNN/DEC-KM approach, in addition to being evaluated on substantially larger networks (200 and 500 nodes versus 50 nodes).

Overall, these recent studies demonstrate the growing importance of hybrid intelligent routing algorithms for energy-efficient WSNs. Nevertheless, most existing approaches rely on optimization heuristics, bio-inspired algorithms, or security enhancements without incorporating lightweight online learning mechanisms that continuously adapt CH selection according to real-time network conditions. Motivated by this research gap, the proposed RL-ILEACH protocol integrates a lightweight tabular Q-learning algorithm into the ILEACH framework, enabling adaptive, experience-driven CH selection while maintaining low computational complexity suitable for resource-constrained Wireless Sensor Networks.

### Qualitative comparison of machine learning-based clustering protocols

A comprehensive qualitative comparison against other prominent machine learning paradigms in WSNs is summarized in Table 1. While deep reinforcement learning (DRL) frameworks and multi-agent reinforcement learning (MARL) approaches offer exceptional adaptability to high-dimensional, continuous state spaces, they inherently introduce substantial computational and communication penalties. DRL-based methods rely on complex deep neural networks that necessitate intense training, high-capacity hardware, and expansive experience replay buffers—requirements that far exceed the physical and battery constraints of traditional, resource-constrained sensor nodes. Similarly, MARL schemes often suffer from high signaling and message overhead due to frequent inter-agent state exchanges and joint-action coordination. In contrast, the proposed RL-ILEACH protocol strikes an optimal balance between intelligence and resource conservation. By utilizing a lightweight, tabular Q-learning model, it achieves real-time, experience-based adaptability to dynamic network topologies and energy fluctuations via minor tabular lookups. This design ensures that the protocol provides the self-learning benefits of advanced artificial intelligence without incurring the prohibitive computational, memory, and energy overhead typical of deep or fully decentralized multi-agent architectures, rendering it highly suitable for practical, long-term heterogeneous WSN deployments.

**Table 1:** Qualitative comparison of machine learning-based clustering protocols.

Metric/feature	Conventional LEACH/ILEACH	Proposed RL-ILEACH	Machine Learning WSN Protocols (K-Means/K-NN/DEC)	Deep RL (DRL/DQN) Approaches	Multi-Agent RL (MARL) Approaches
Decision Mechanism	Static Formulae/Heuristics	Tabular Q-Learning (Experience-based)	Supervised/ Unsupervised Classification& Partitioning	Deep Neural Network Function Approx.	Cooperative/Decentralized Agents
Computational Overhead	Ultra-Low	**Low** (Small tabular lookups)	Low-to-Medium (Distance calculation/classification)	High (Requires intense training/inference)	Medium-High (Depends on state-sharing)
Signaling/Message Overhead	Low	**Low**	Low to Medium	Medium	High (Frequent inter-agent communication)
Adaptability to Topology	Poor (Static thresholds)	**High** (Dynamic state adjustments)	Moderate (Requires periodic re-clustering/retraining)	Very High (Handles continuous states)	Extreme (Adapts to large-scale swarms)
Ideal Deployment Environment	Stationary, homogeneous WSNs	**Dynamic**,** resource-constrained heterogeneous WSNs**	Static or Semi-Static WSNs	Base station-driven or edge-computed WSNs	Large-scale, highly mobile ad-hoc networks

## Proposed RL-ILEACH protocol

The features and constraints of current clustering protocols for WSNs were examined in the preceding section, with a focus on the (ILEACH) protocol. ILEACH’s cluster head selection process is still mostly reliant on predetermined heuristic rules and static decision criteria, despite the fact that it improves energy efficiency when compared to the traditional LEACH protocol through better cluster formation and communication tactics. As a result, the protocol cannot successfully adjust to the constantly shifting circumstances of WSNs, including changes in residual energy, node density, communication distance, traffic load, and network architecture. Particularly in large-scale and dynamic IoT-enabled systems, these constraints frequently lead to imbalanced energy consumption, early fatigue of crucial sensor nodes, decreased network stability, and a shorter overall network lifetime.

The proposed RL-ILEACH framework consists of multiple sequential stages: first, sensor nodes gather local network information and exchange the control messages needed for state construction; next, the reinforcement learning agent assesses the current state and calculates the expected long-term benefit associated with each possible action; after the CH selection process, ordinary sensor nodes are allowed to associate with the most appropriate CH based on communication cost and signal quality; CHs then perform data aggregation to minimise redundant transmissions before sending the aggregated information to the BS using energy-aware communication. The RL agent can adjust its strategy for subsequent rounds by updating the network state at the conclusion of each communication cycle based on the remaining energy levels, communication results, and traffic circumstances. The protocol can dynamically adjust to shifting network conditions thanks to this ongoing learning process, which eliminates the need for manually adjusted parameters or set thresholds. The proposed RL-ILEACH protocol is new because it substitutes an adaptive reinforcement learning framework that can optimise CH election based on real-time network circumstances for the static CH selection method. The suggested protocol achieves a better balance in energy consumption among sensor nodes, minimises needless communication overhead, enhances cluster stability, extends the stability period before the first node dies, and increases the lifetime of the wireless sensor network by taking into account multiple network metrics at once and continuously updating the decision policy through environmental feedback. Additionally, RL-ILEACH offers a more reliable and scalable solution than traditional clustering techniques because of the adaptive nature of the learning process, which makes it more appropriate for dynamic IoT applications where network topology and traffic patterns regularly change.

### Radio energy model

**Fig. 1:**
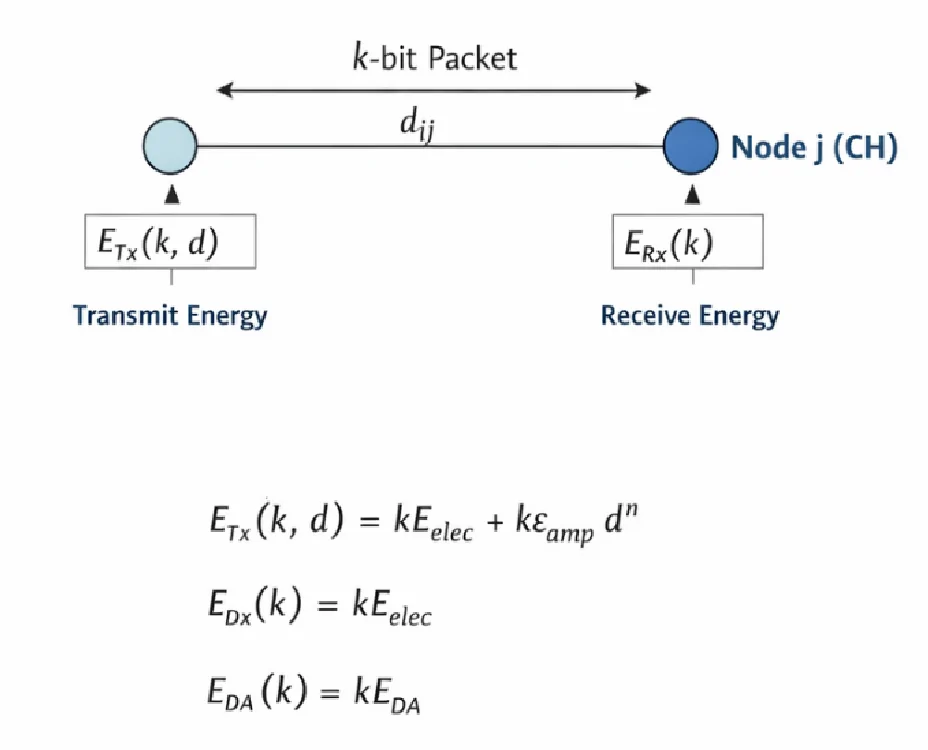
Radio energy consumption model.

The proposed RL-ILEACH protocol adopts the first-order radio energy model commonly used in WSN research. Each sensor node consumes energy during data transmission, reception, and aggregation Fig. 1.

1 $$\:{E}_{Tx}(k,d)=\left\{\begin{array}{c}k\:\left({E}_{elec}+{E}_{fs}{d}^{2}\right),\:\:\:\:\:\:\:\:d\le\:\:{d}_{0,}\:\\\:k\:\left({E}_{elec}+{E}_{mp}{d}^{4}\right),\:\:\:\:\:\:\:\:d>\:{d}_{0,}\end{array}\right.$$

where:

- $\:{E}_{elec}$ is the per-bit energy required for the transmitter/receiver circuitry,
- $\:{E}_{fs},\:{E}_{mp}\:$represent the amplifier energy parameters for free-space and multipath fading channels, respectively,
- $\:{d}_{0,}$ is the threshold distance that distinguishes between the two propagation models.

2 $$\:{d}_{0}=\sqrt{\raisebox{1ex}{${E}_{fs}$}\!\left/\:\!\raisebox{-1ex}{${E}_{mp}$}\right.}$$

The energy consumed to receive *k* bits is defined as^21^:

3 $$\:{E}_{Rx}=k*\:{E}_{elec}$$

Data Aggregation Energy:

4 $${\mathrm{E}}\_{\mathrm{DA}}\left( {\mathrm{k}} \right){\text{ }}={\text{ E}}\_{\text{DA }} \times {\text{ k}}$$

where k is the packet size in bits, d is the transmission distance, E_elec is the electronic energy, and E_amp / E_mp represent amplifier energy factors^22–25^.

### Reinforcement learning model

By incorporating Reinforcement Learning for CH selection, the suggested RL-ILEACH model adds intelligence and flexibility to the ILEACH methodology. The primary goal of this enhancement is to get over the drawbacks of static CH selection and provide a self-learning, energy-conscious, and scalable routing protocol that can increase overall performance and prolong the lifetime of Wireless Sensor Networks. In this study, the Q-learning algorithm was implemented using a learning rate (η) of 0.1 and a discount factor (λ) of 0.9. The exploration parameter ε was initially set to 0.1 and gradually reduced during the learning process to encourage early exploration and later exploitation.

$$\:\epsilon\:\left(r\right)=\mathrm{m}\mathrm{a}\mathrm{x}({\epsilon\:}_{min},{\epsilon\:}_{0}{e}^{-kr})$$

where * r*denotes the current simulation round, $\:{\epsilon\:}_{\mathrm{m}\mathrm{i}\mathrm{n}}=0.01$is the minimum exploration rate, and * k=0.001*is the decay coefficient. This schedule enables sufficient exploration during the early stages of training while gradually favoring exploitation of the learned policy as convergence is approached.

The reward function coefficients were empirically determined through preliminary simulations. The values α = 0.5, β = 0.3, and γ = 0.2 were selected because they provided the best balance among energy efficiency, throughput, and packet delivery ratio. Since extending network lifetime is the principal objective of RL-ILEACH, residual energy was assigned the largest weight. Sensitivity analysis revealed that the proposed protocol maintained stable performance over a reasonable range of parameter variations, confirming the robustness of the reward formulation.

State Space: Residual energy, node degree, distance to BS, previous CH role.

Action Space: CH selection (CH/Non-CH).

Reward Function:

$$\begin{aligned} {\mathrm{Reward}}\, & {\text{ = }}\,\alpha {\text{ }} \times {\text{ }}\left( {{\text{Residual Energy}}} \right){\text{ }} \\ & {-}{\text{ }}\beta {\text{ }} \times {\text{ }}\left( {{\text{Transmission Cost}}} \right){\text{ + }}\gamma {\text{ }} \times {\text{ }}\left( {{\text{Successful Data Delivery}}} \right) \\ \end{aligned}$$

The proposed RL-ILEACH employs a fully distributed tabular Q-learning mechanism, where each sensor node maintains and updates its own local Q-table using only locally available information (residual energy, neighbor density, distance to the BS, and previous CH status). No centralized controller or global state information is required during the learning process. The proposed reinforcement learning mechanism introduces minimal communication overhead because all learning operations are performed locally. No additional control packets are exchanged for Q-table updates, and no global information dissemination is required. The protocol only utilizes the control messages already employed during cluster formation and routing, such as advertisement, join-request, and scheduling packets.

#### Q-table size

**Fig. 2:**
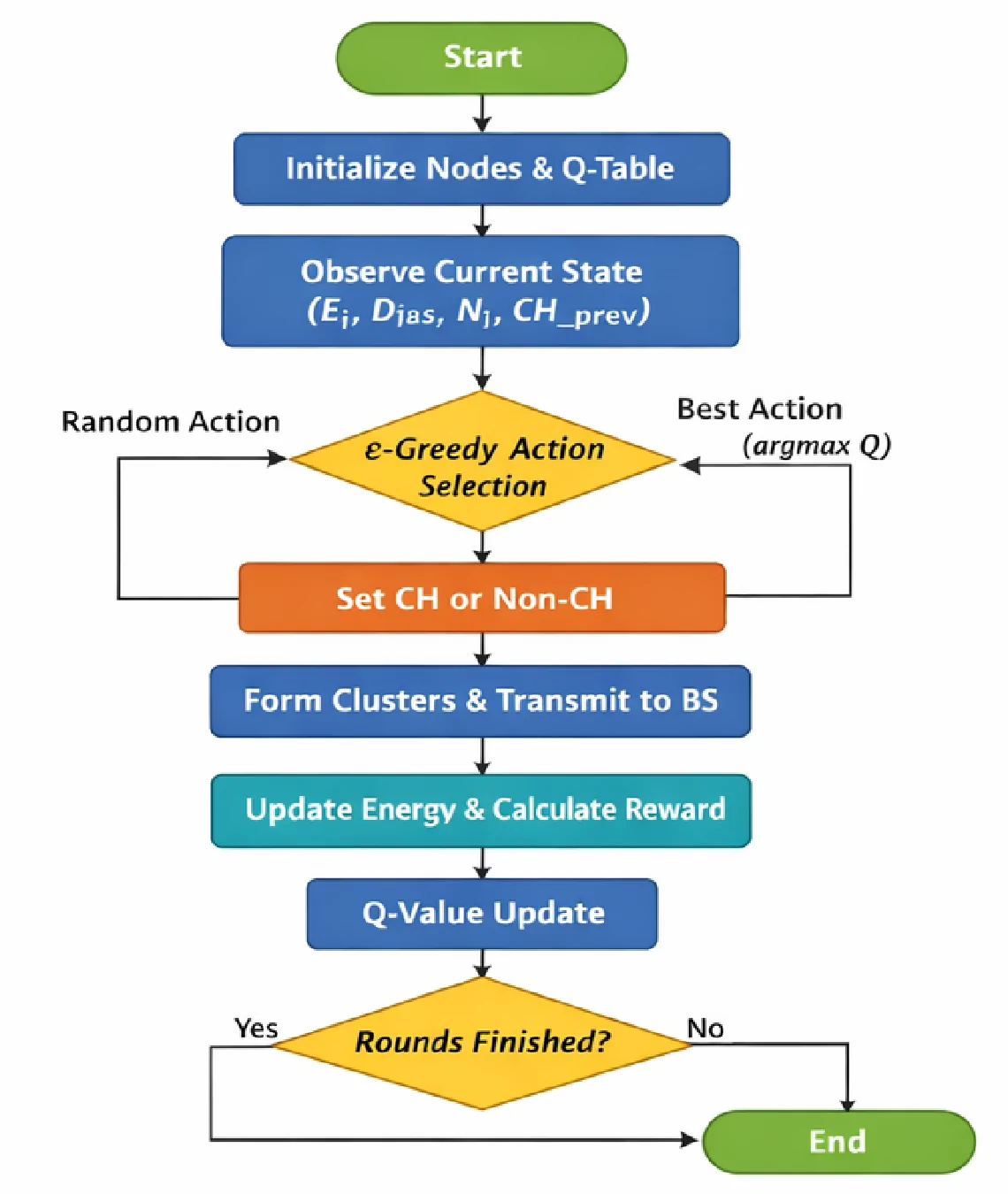
Proposed reinforcement learning-ILEACH.

Since the proposed RL-ILEACH employs tabular Q-learning, continuous state variables are discretized before learning. Residual energy and distance to the base station are uniformly quantized into 10 levels each, node degree into 5 levels, while the previous cluster-head status is represented by two binary states. Consequently, the overall state space consists of $\:10\times\:10\times\:5\times\:2=1000$discrete states. Considering the two available actions (Cluster Head and Non-Cluster Head), the resulting Q-table contains $\:1000\times\:2=2000$state-action entries. This compact table requires only approximately 16 KB of memory using double-precision storage, making the proposed RL-ILEACH computationally lightweight and suitable for resource-constrained wireless sensor nodes (Fig. 2).

### Pseudo-code for the RL-ILEACH protocol

**Figure:**
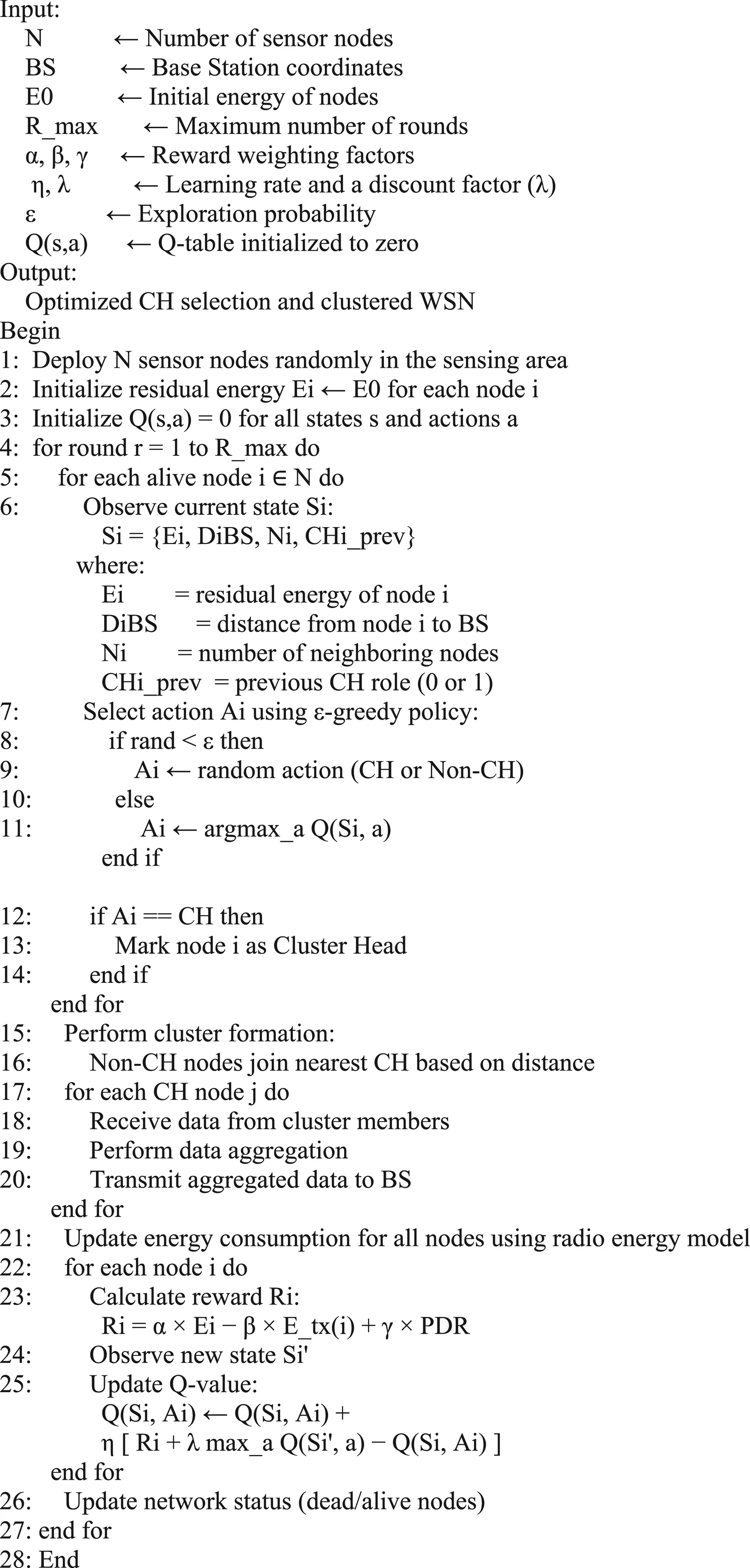

### Security considerations and cybersecurity aspects

Wireless Sensor Networks are susceptible to a variety of security threats due to their distributed architecture, limited computational resources, and wireless communication medium. Common attacks include sinkhole attacks, where a malicious node attracts surrounding traffic by advertising an attractive routing path; blackhole attacks, in which compromised nodes intentionally drop received packets; wormhole attacks, where two colluding malicious nodes establish a low-latency tunnel to disrupt routing; Sybil attacks, in which a malicious node assumes multiple fake identities; and selective forwarding attacks, where only selected packets are discarded to degrade network performance.

The proposed RL-ILEACH protocol primarily focuses on improving energy efficiency and network lifetime rather than providing a dedicated security mechanism. Nevertheless, the reinforcement learning framework offers opportunities for enhancing routing security. Since cluster-head selection is based on dynamic state observations and reward optimization, additional security-related metrics, such as node trust, packet forwarding behavior, or anomaly scores, can be incorporated into the state representation and reward function. Consequently, nodes exhibiting suspicious behavior or low trust values can be assigned lower rewards, reducing their probability of being selected as cluster heads.

Although security mechanisms were not explicitly implemented in this study, integrating trust management or lightweight intrusion detection with the proposed reinforcement learning framework represents an important direction for future research. Such integration could simultaneously improve network resilience, energy efficiency, and secure routing in dynamic Wireless Sensor Network environments.

## Experimental results and discussion

The simulation results used to assess the effectiveness of the suggested RL-ILEACH methodology are shown and discussed in this section. Key performance parameters, including as energy consumption, number of living nodes, stability period, packet delivery ratio (PDR), and network longevity, are used to compare the efficacy of RL-ILEACH with traditional LEACH and ILEACH protocols. To provide an impartial and fair comparison, every simulation was run under the same network circumstances.

### Simulation setup

All simulations and expe rimental procedures for this research were executed using MATLAB software on a system operating under Windows 10 Enterprise. The hardware setup included an Intel Core i7-9750 H processor clocked at 2.60 GHz and equipped with 16 GB of RAM (Table 2).

**Table 2:** Simulation parameters.

Symbol	Parameters	Values
X_m_, y_m_	Network Area	200*200
N	Number of Nodes	200, 500
P	Cluster head probability	0.1
E_0_	Energy for each node	0.5 j
E_TX_	transmitter energy	50*0.000000001
E_RX_	receiver energy	50*0.000000001
E_DA_	Aggregation Energy	5*0.000000001
E_amp_	amplification energy	0.0013*0.000000000001
rmax	Number of Rounds	4000
d_0_	distance	87 m

**Fig. 3:**
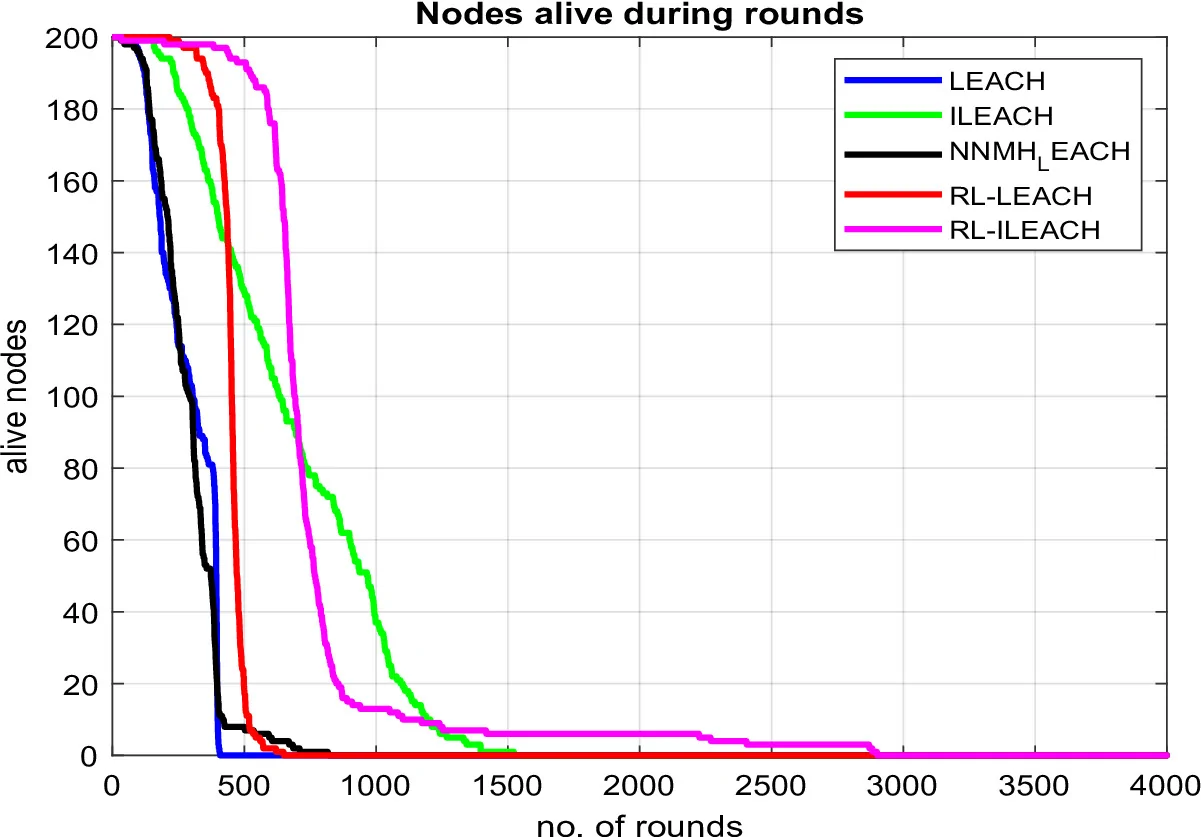
Comparison of the number of alive nodes versus simulation rounds for LEACH, ILEACH, NNMH-LEACH, RL-LEACH, and the proposed RL-ILEACH under a 200-node network scenario.

Network stability may be directly determined by counting the number of nodes that remain active during simulation cycles. The findings show that, in comparison to LEACH and ILEACH, RL-ILEACH sustains a greater number of active nodes for a longer period of time. Due to random CH assignment, certain nodes in LEACH incur early node deaths and premature energy depletion. By enhancing CH selection, ILEACH prolongs the stability period, but it is still not flexible when node energy levels and topology fluctuate. As demonstrated in Figs. 3 and 10 LEACH exhibited the fastest degradation, with all sensor nodes becoming inactive after approximately 450 rounds due to its random CH selection mechanism. RL-LEACH delayed node depletion compared with LEACH; however, all nodes died before 800 rounds. Similarly, ILEACH improved the stability period through energy-aware CH selection, but complete network depletion occurred around 1,350 rounds. NNMH-LEACH achieved a longer lifetime than LEACH and RL-LEACH, maintaining active nodes until approximately 2,000 rounds.

In contrast, the proposed RL-ILEACH maintained a significantly higher number of alive nodes throughout the simulation. Approximately 270 nodes remained active after 1,200 rounds, while the competing protocols had already experienced severe node depletion. Furthermore, RL-ILEACH preserved nearly 190 alive nodes at 2,300 rounds, around 150 alive nodes at 3,000 rounds, and more than 120 sensor nodes remained operational even after 4,000 rounds. At this stage, all other protocols had already reached complete network failure.

**Fig. 4:**
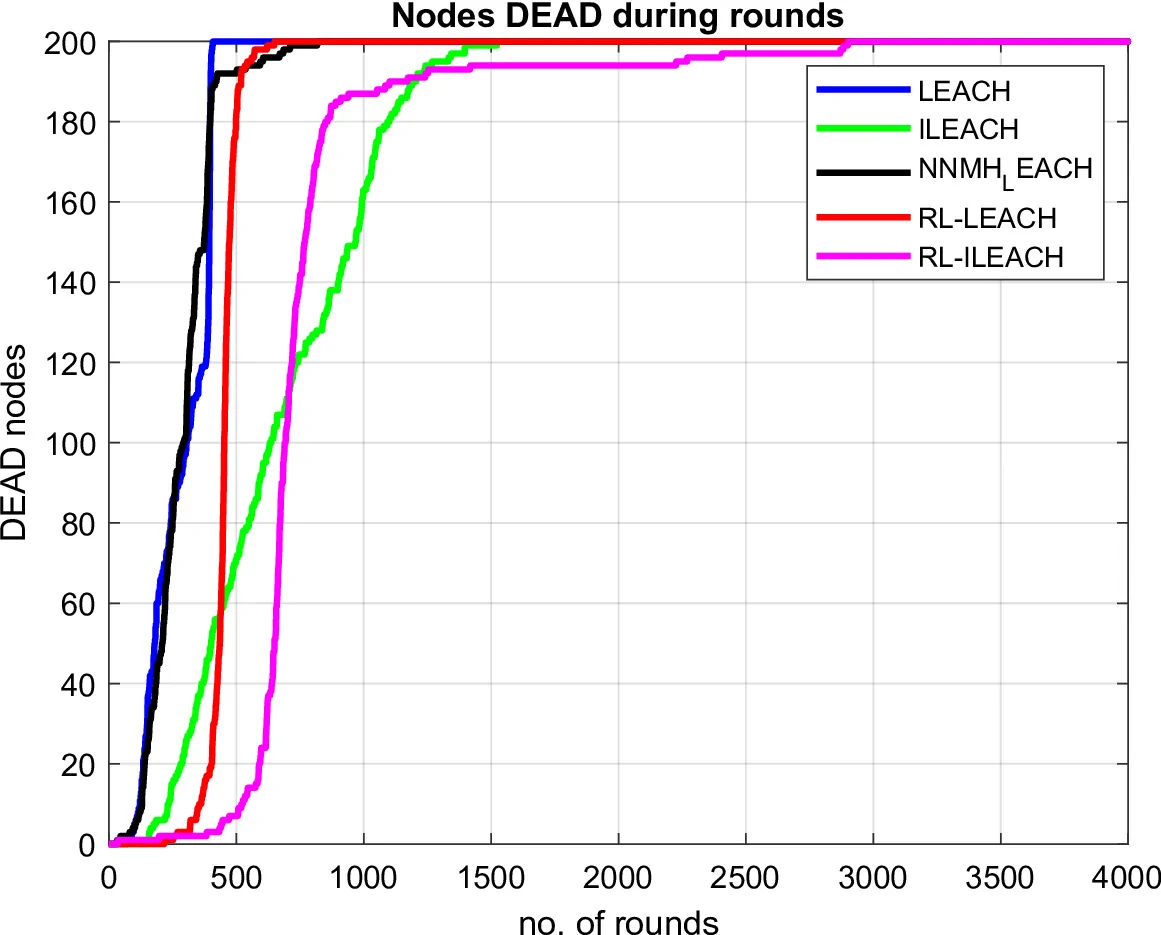
Comparison of the number of dead nodes versus simulation rounds for LEACH, ILEACH, NNMH-LEACH, RL-LEACH, and the proposed RL-ILEACH under a 200-node network scenario.

RL-ILEACH outperforms all other protocols in terms of network lifespan, which is defined as the number of rounds until the final node in the network is depleted. While ILEACH offers a minor improvement and LEACH has the shortest lifespan due to wasteful energy use, RL-ILEACH beats both by cleverly adjusting CH selection choices throughout network operation. The learning-based technique maximizes the network’s operational lifespan by allowing RL-ILEACH to react to energy fluctuations and topological changes. This significant increase in network lifespan demonstrates that combining ILEACH with reinforcement learning provides a reliable and scalable solution for long-term WSN installations.: The Last Node Dies (LND) metric defines the absolute operational boundary of the network. As demonstrated in Fig. 4 LEACH and RE-LEACH collapse completely by rounds 456 and 634 respectively. While ILEACH and NN-MH-LEACH sustain network operations longer (1785 and 1969 rounds), RL-ILEACH preserves active routing paths with 12 alive nodes remaining even at the maximum simulation boundary of 4000 rounds. This proves that tabular Q-learning prevents the localized “energy holes” common in static heuristic protocols.

**Fig. 5:**
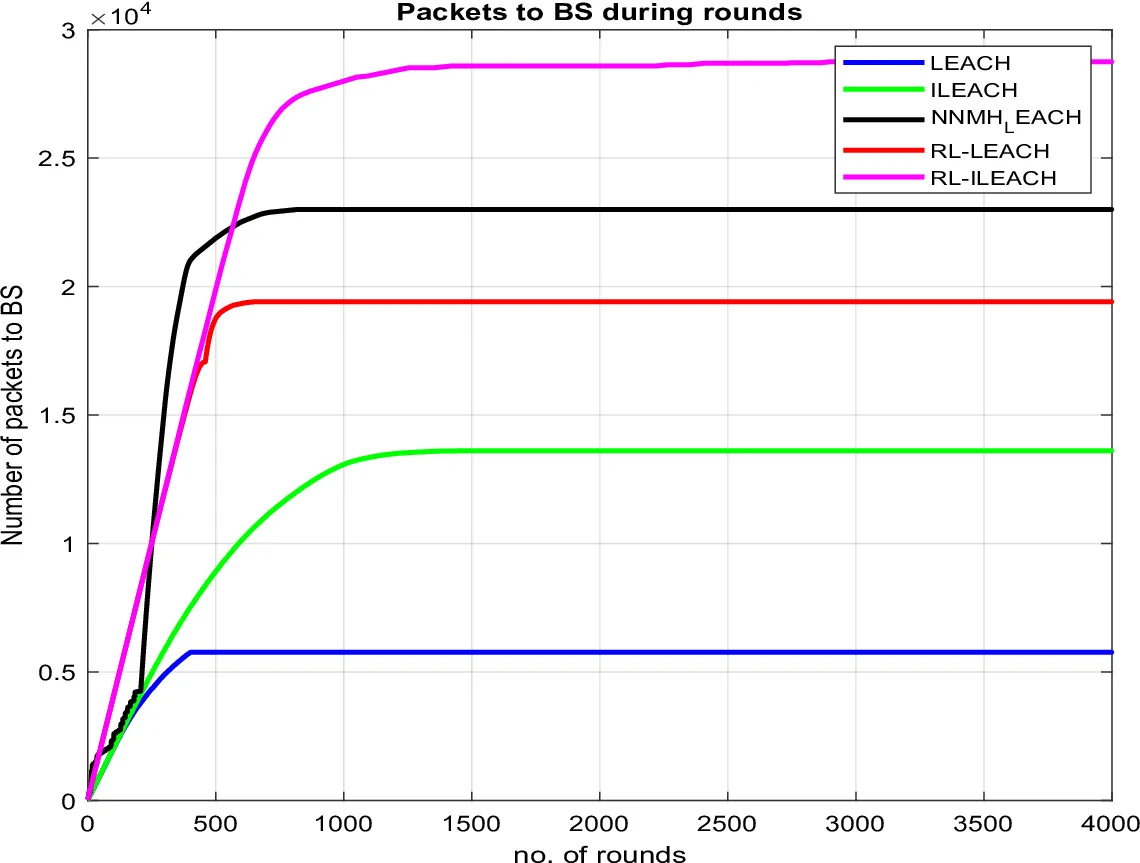
The packet to BS versus simulation rounds for 200 nodes network.

**Fig. 6:**
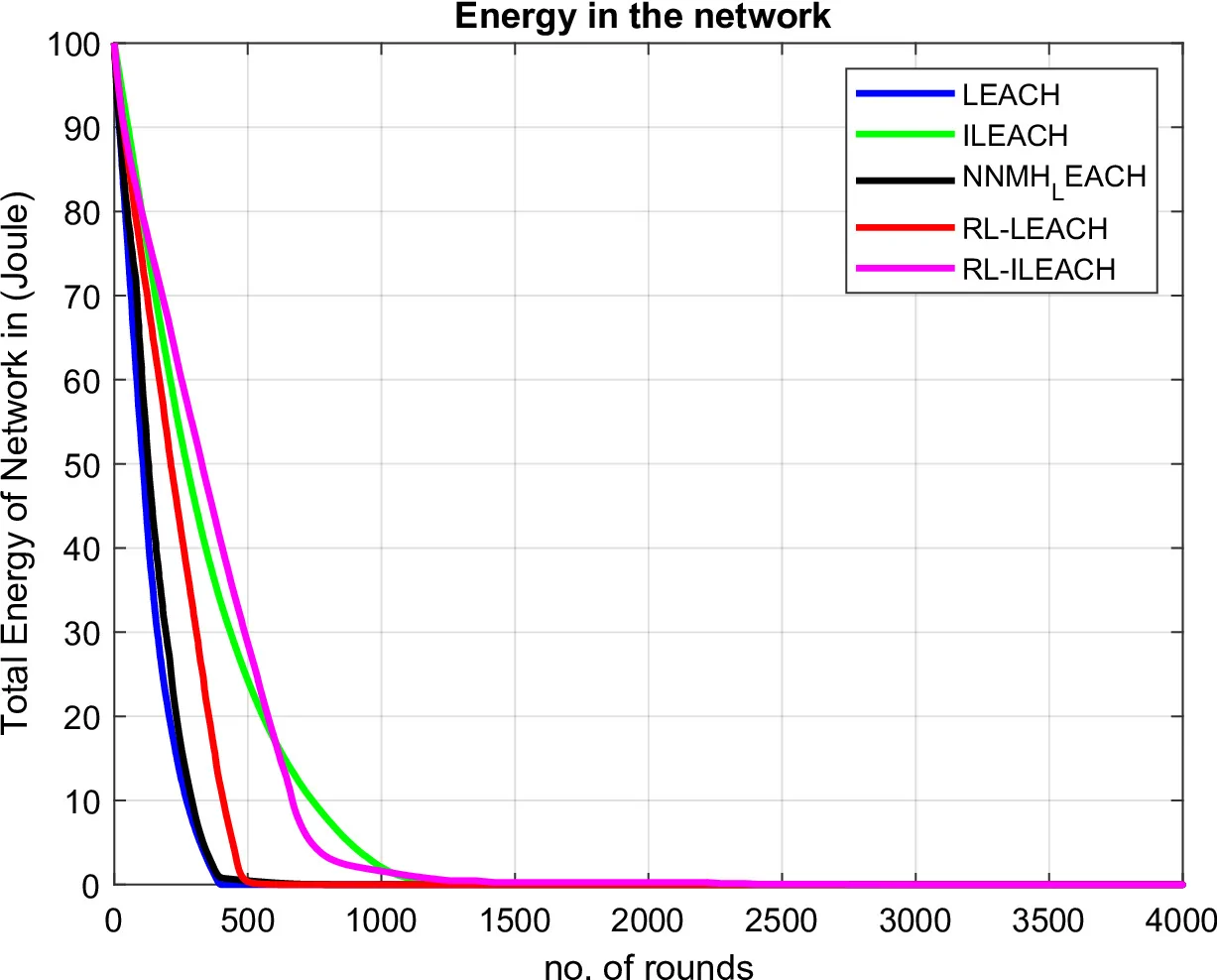
Total network energy consumption as a function of simulation rounds for the evaluated clustering protocols for 200 nodes network.

Figure 5 presents the number of packets successfully transmitted to the base station, reflecting the communication efficiency and data collection capability of each routing protocol. Figure 6 compares the total residual energy of the network throughout the simulation for the evaluated clustering protocols under a 200-node deployment. This experiment assesses the energy efficiency of each routing protocol by monitoring the rate of energy depletion during network operation. A slower energy decay indicates better load balancing and more efficient utilization of the available energy resources.

As Fig. 6 the effectiveness and sustainability of wireless sensor networks are significantly influenced by energy consumption. The findings unequivocally demonstrate that, in comparison to both LEACH and ILEACH, the suggested RL-ILEACH methodology uses substantially less energy over subsequent cycles. Low-energy nodes are frequently designated as CHs in LEACH due to random CH selection, which causes quick energy depletion. ILEACH overcomes this by taking distance and residual energy into account, however under dynamic network conditions, its static threshold-based decision-making still results in unequal energy dissipation. By dynamically learning appropriate CH selection rules based on real-time network conditions, RL-ILEACH, on the other hand, delivers higher energy efficiency. In order to minimize unnecessary energy consumption during both intra-cluster and inter-cluster communication, the reinforcement learning agent avoids choosing low-energy or poorly positioned nodes as CHs. As a result, RL-ILEACH’s total energy decay rate is significantly slower, demonstrating its efficacy in energy-aware clustering.

**Fig. 7:**
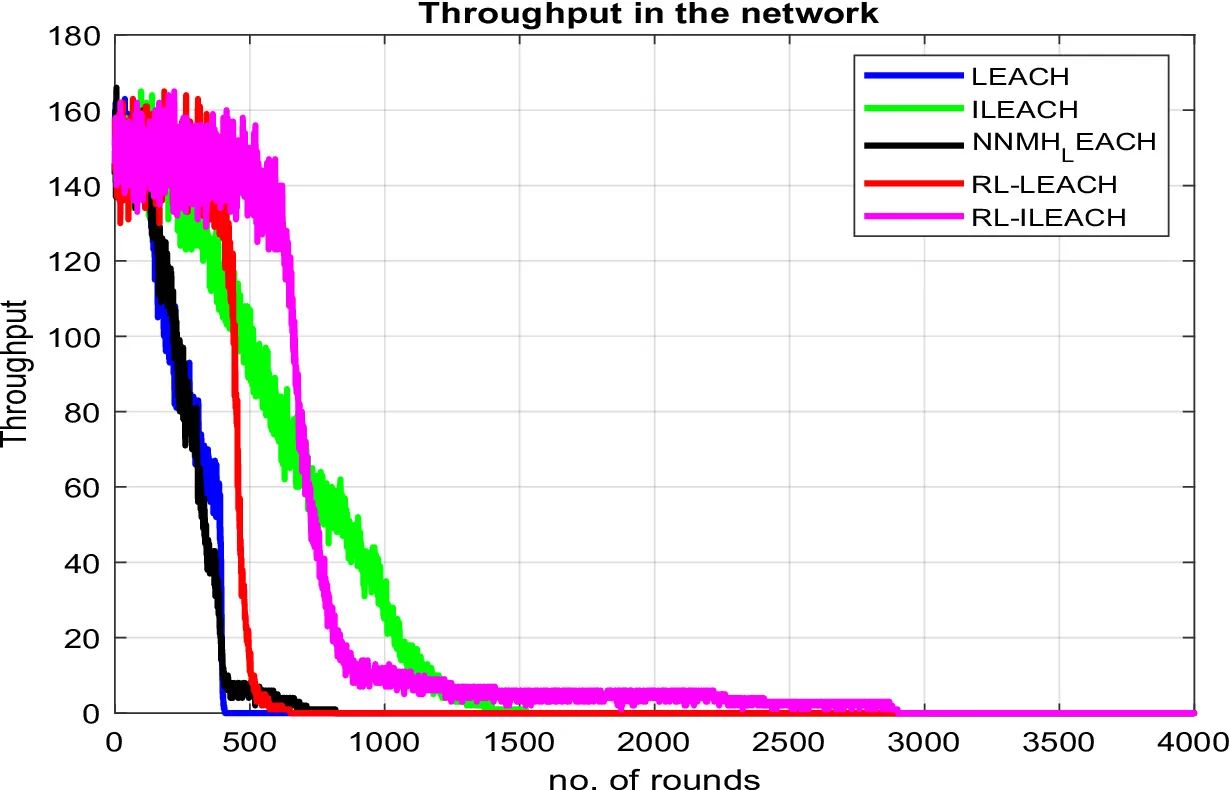
The throughput of network versus simulation rounds for 200 nodes network.

**Fig. 8:**
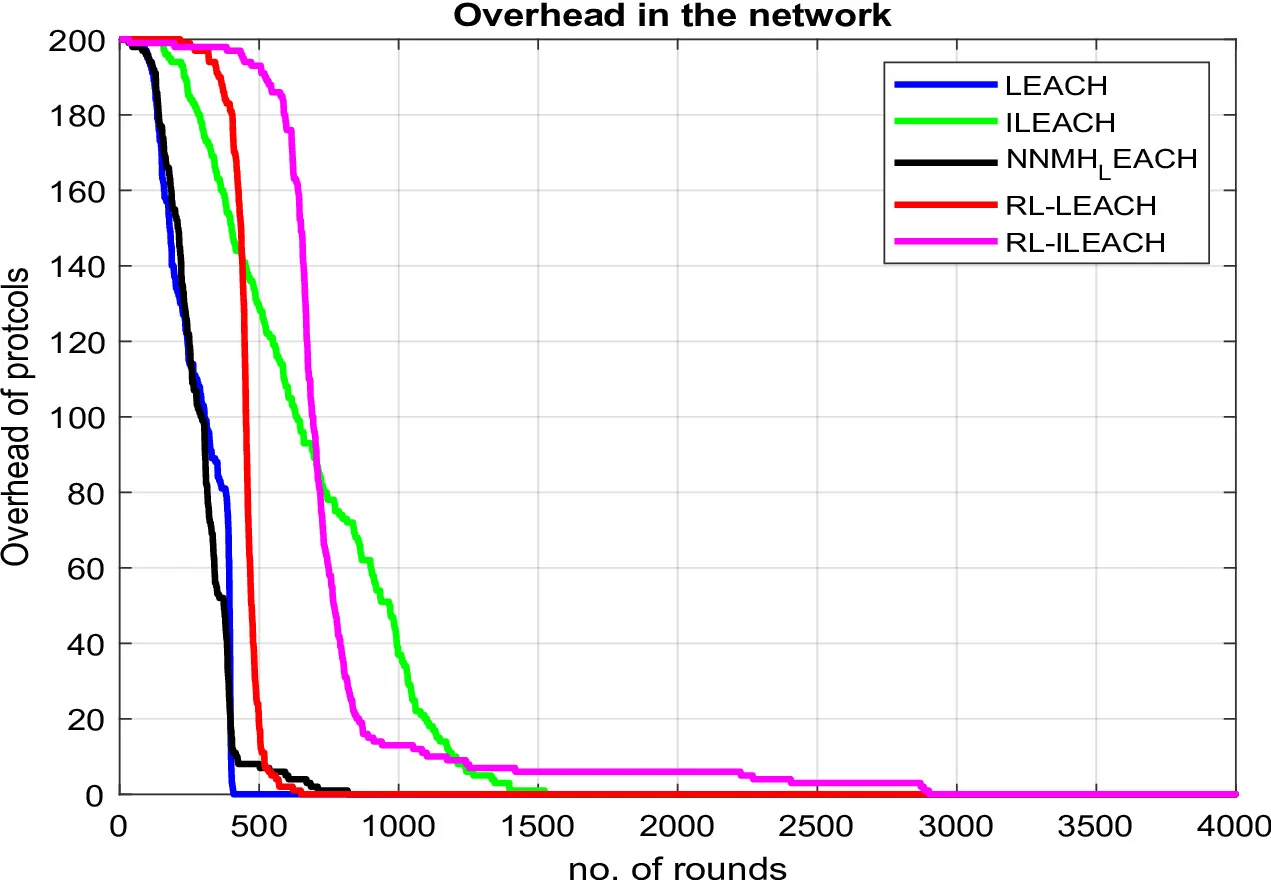
The overhead as a function of simulation rounds for the evaluated clustering protocols under a 200-node network scenario.

**Fig. 9:**
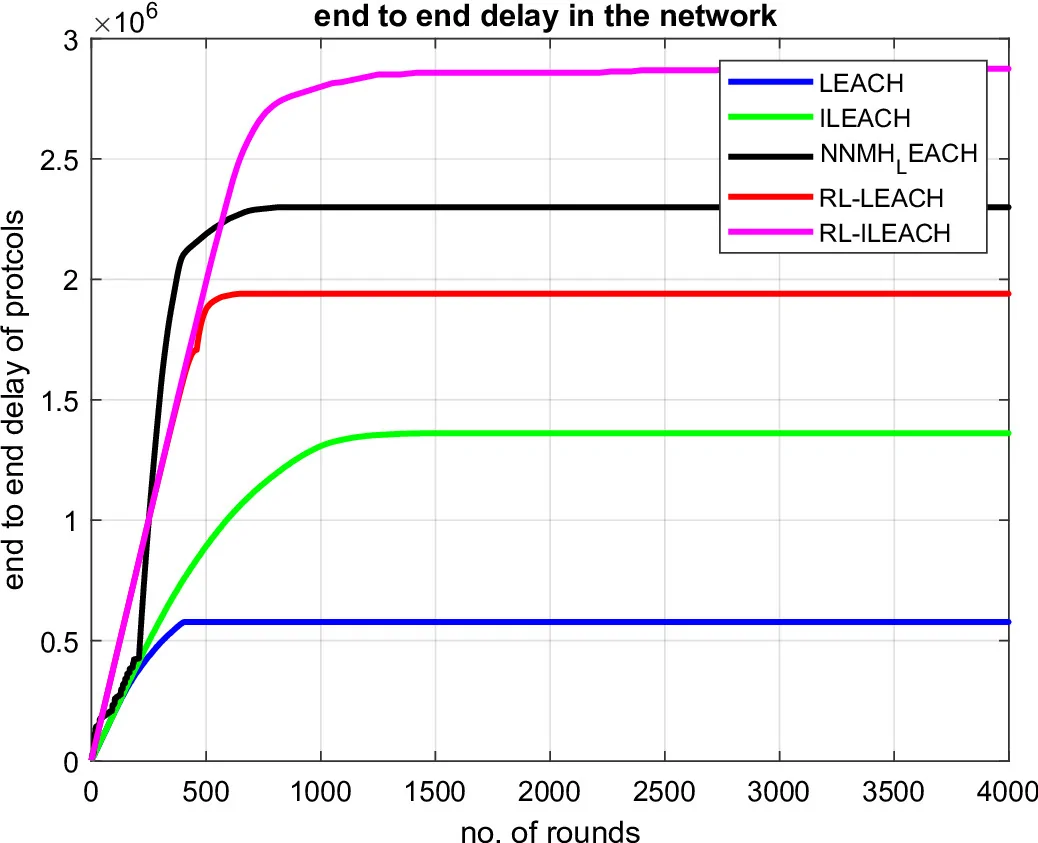
The end to end dealy comparsion of network under a 200-node network scenario.

Figure 7 illustrates the network throughput achieved by the compared protocols over the simulation rounds. Throughput represents the successful delivery of data packets to the base station and serves as an important indicator of communication efficiency and routing reliability. Figure 8 presents the communication overhead generated by each clustering protocol. This experiment evaluates the amount of control information exchanged during cluster formation and routing operations, where lower overhead reflects more efficient network management and reduced energy consumption. Figure 9 compares the end-to-end delay of the evaluated protocols under the 200-node network scenario. This metric measures the average time required for data packets to travel from sensor nodes to the base station and reflects the responsiveness and communication efficiency of the routing protocol.

The results of the various simulations are similar to those of the first simulation, in which we alter the base station’s diameter using the following data: xm = 200; ym = 200; sink.x = xm/2; sink.y = ym/2; *n* = 200; *p* = 0.1. Table 3 explains the stages of dead nodes and how our suggested RL-LEACH protocol outperforms the compared protocol.

Additionally, our RL-ILEACH protocol performed best when the hop count and probability were changed in various simulations. Changes in the geographic size of the network also produced incredible results for our protocol, which is very good.

**Table 3:** FND, HND and LND for dead nodes compared protocols.

	FND	HND	LND
LEACH	156	441	456
ILEACH	936	1073	1785
NNMH_LEACH	131	480	1969
RE-LEACH	257	504	634
RE-ILEACH	524	990	188 dead nodes at 4000

The findings of the most recent simulation, which was conducted in a dense network with 500 nodes in the same simulation diameter as the first, are listed below.

**Fig. 10:**
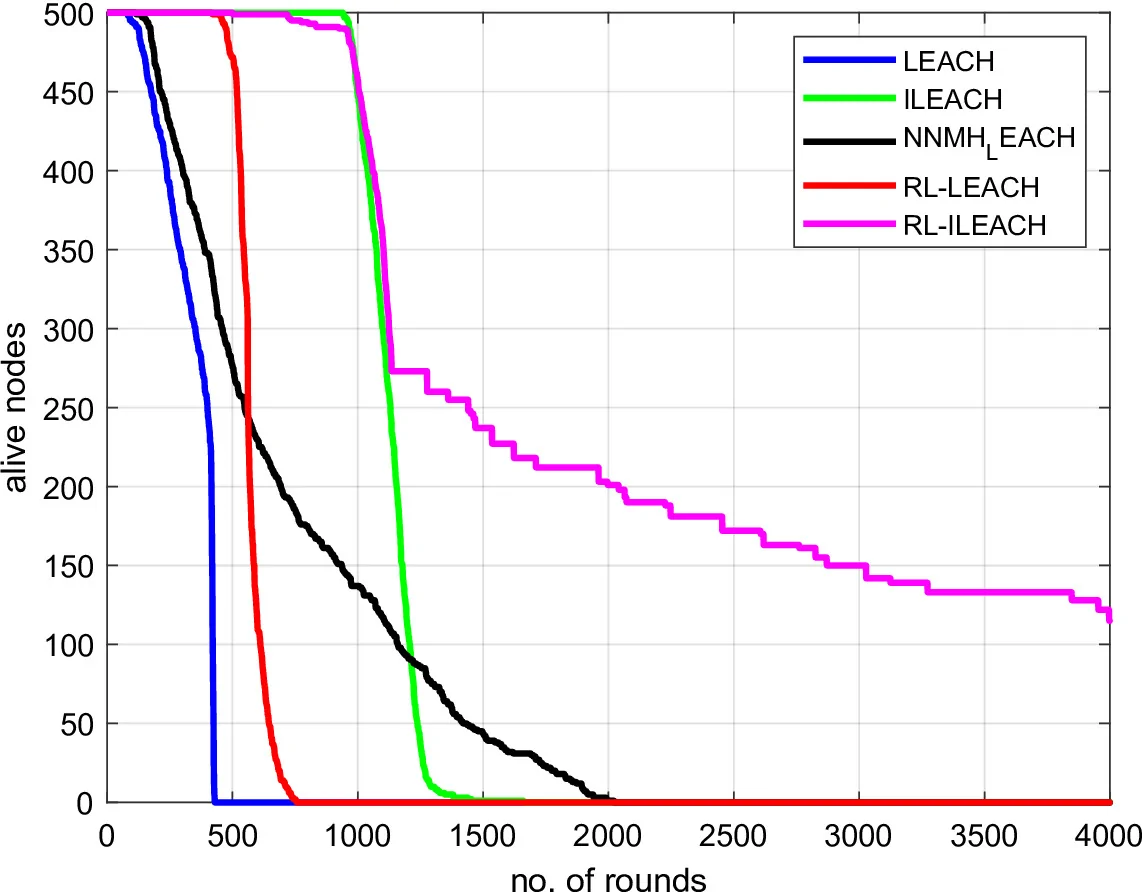
Comparison of the number of alive nodes versus simulation rounds for LEACH, ILEACH, NNMH-LEACH, RL-LEACH, and the proposed RL-ILEACH under dense network scenario (500 nodes network).

**Fig. 11:**
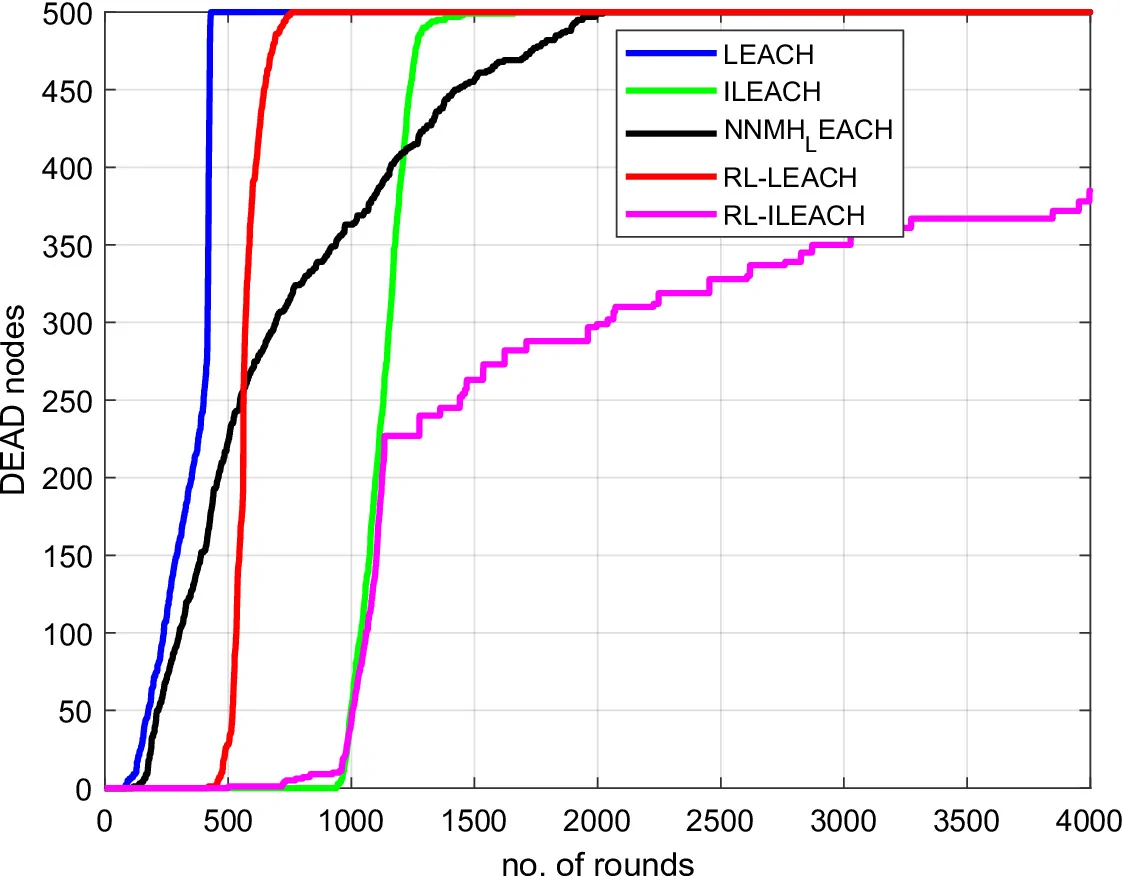
Comparison of the number of dead nodes versus simulation rounds for LEACH, ILEACH, NNMH-LEACH, RL-LEACH, and the proposed RL-ILEACH under dense network scenario (500 nodes network).

To evaluate the scalability of the proposed approach, a second experiment was conducted using a dense wireless sensor network consisting of 500 sensor nodes. Figure 10 compares the number of alive nodes over the simulation rounds, illustrating the ability of each protocol to maintain network stability under high node density. Figure 11 illustrates the progression of dead sensor nodes in the dense network scenario. The comparison demonstrates the effectiveness of the routing protocols in balancing energy consumption and delaying node failures as network density increases.

The results presented in Fig. 11 clearly demonstrate the superiority of the proposed RL-ILEACH protocol in extending the operational lifetime of the Wireless Sensor Network. Compared with the conventional LEACH protocol, RL-ILEACH prolonged the network lifetime from approximately 450 rounds to 4000 rounds, representing an improvement of about 784%. RL-ILEACH outperformed ILEACH by increasing the network lifetime from around 1,350 rounds to 4,000 rounds, corresponding to an improvement of approximately 130%. Moreover, relative to NNMH-LEACH, which sustained network operation for nearly 2,000 rounds, RL-ILEACH achieved a further 108% increase in lifetime. These remarkable improvements confirm that the reinforcement learning-based CH selection mechanism effectively distributes the energy burden among sensor nodes, avoids premature node depletion, and maintains a larger number of active nodes throughout the simulation period. Consequently, RL-ILEACH provides a more robust and energy-efficient solution for large-scale and dynamic Wireless Sensor Network environments. It is worth noting that the reported lifetime gains of RL-ILEACH (784% over LEACH, 130% over ILEACH, 108% over NNMH-LEACH) are not directly comparable to the percentage improvements reported for DEC-KM over DEC-KNN^20^(e.g., 9.52% FSD, 9.49% PSA, 0.86% energy), since the two studies use different baseline protocols (ILEACH vs. DEC), different network scales (200 to 500 nodes vs. 50 nodes), and different comparison baselines (other RL/NN-based LEACH variants vs. the original DEC protocol). Nonetheless, both works support the broader conclusion that lightweight, non-deep learning techniques, whether an online Q-learning agent or offline distance-based ML methods such as KNN and K-Means, can meaningfully extend WSN lifetime over heuristic clustering baselines without the computational burden of deep or multi-agent reinforcement learning.

**Fig. 12:**
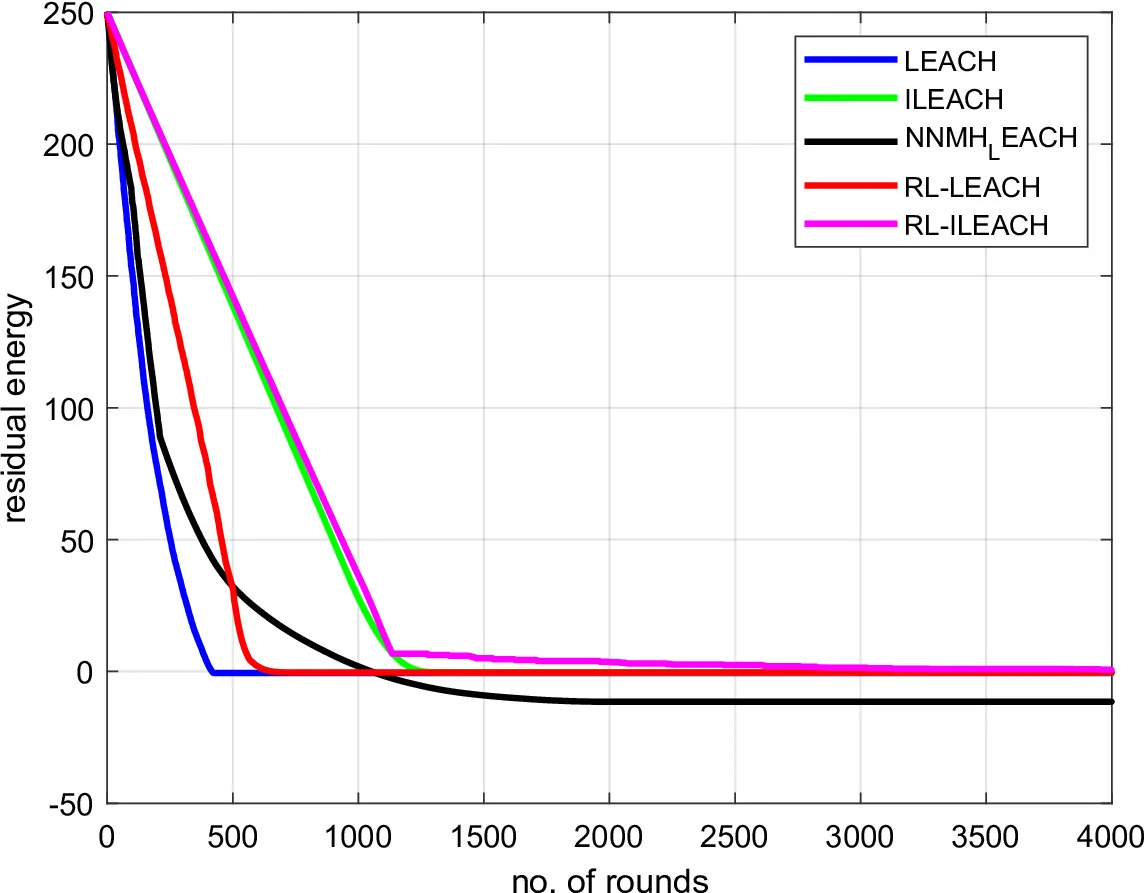
The residual energy as a function of simulation rounds for the evaluated clustering protocols under dense network scenario(500 nodes network).

**Fig. 13:**
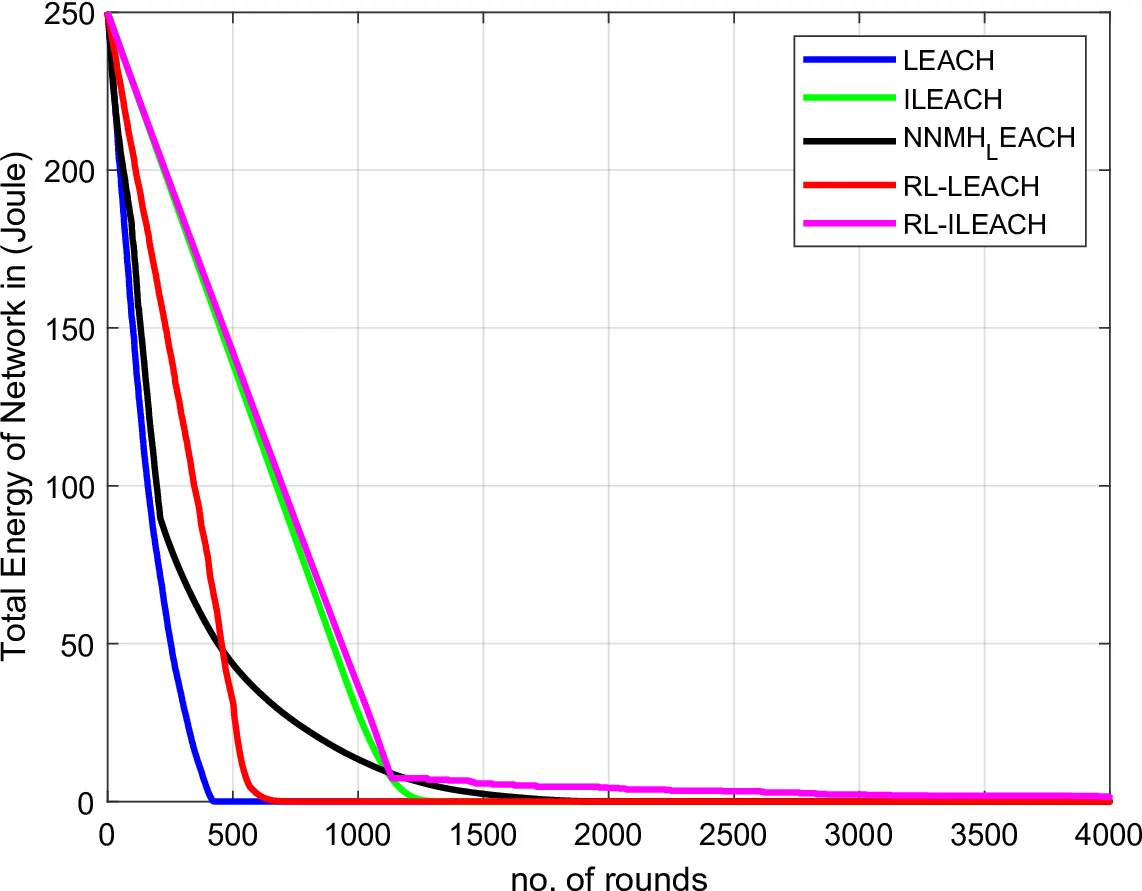
Total network energy consumption as a function of simulation rounds for the evaluated clustering protocols under dense network scenario(500 nodes network).

Figure 12 presents the residual energy of the network throughout the simulation for the dense 500-node deployment. Monitoring the remaining network energy provides insight into the efficiency of the cluster head selection strategy and the overall energy utilization of each protocol. Figure 13 compares the total network energy consumption under the dense deployment scenario. The objective of this experiment is to evaluate how effectively each routing protocol conserves energy while maintaining continuous network operation.

**Fig. 14:**
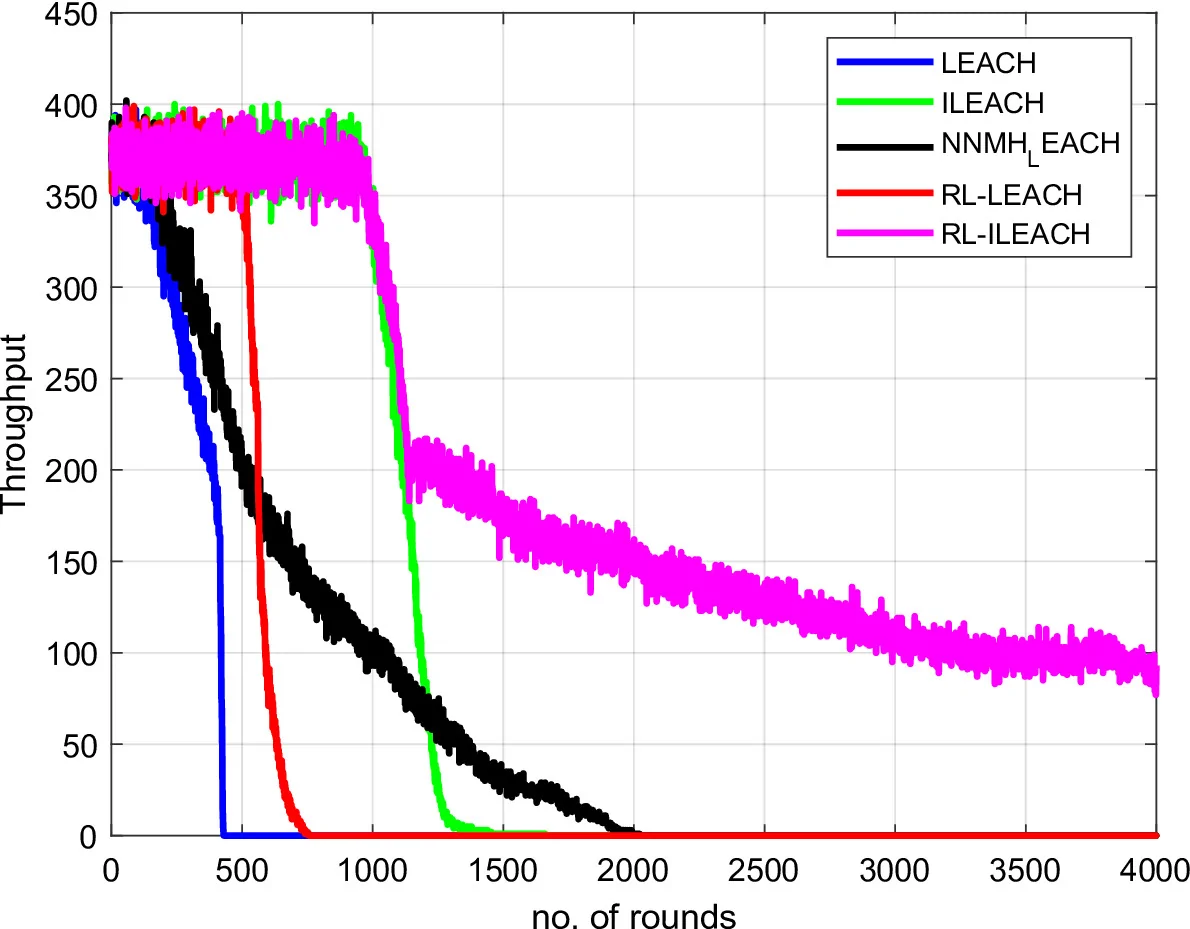
The throughput of network in last simulation under the dense network scenario(500 nodes network).

**Fig. 15:**
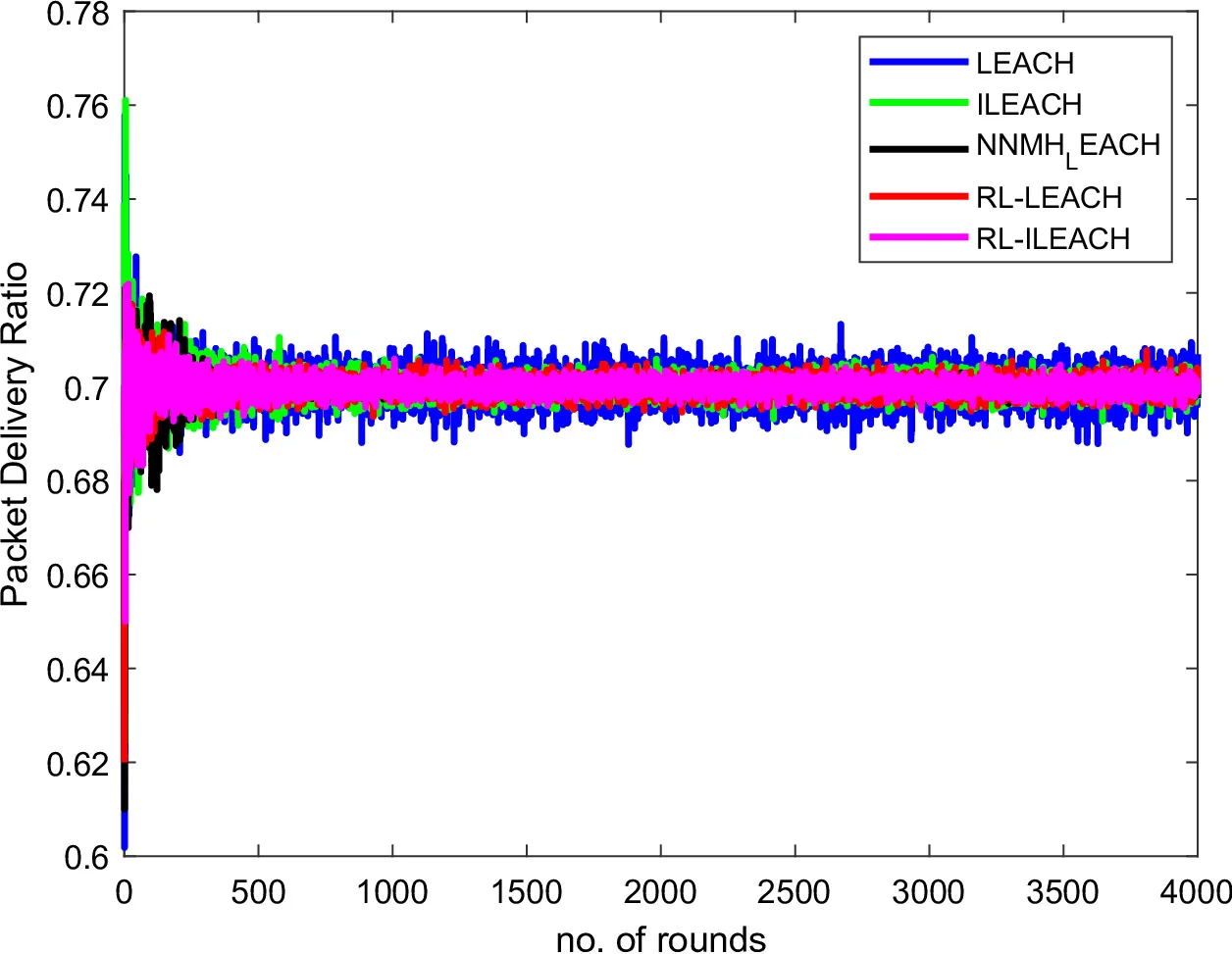
The packet delivery ratio comparison among the evaluated protocols under the dense network scenario(500 nodes network).

Figure 14 illustrates the throughput achieved by the evaluated clustering protocols in the dense network scenario. Higher throughput indicates improved communication reliability and a greater capability to deliver sensed information successfully to the base station. Figure 15 compares the Packet Delivery Ratio (PDR) among the evaluated protocols under the 500-node deployment. The PDR measures the percentage of successfully delivered packets and reflects the reliability and robustness of the proposed routing protocol under increased network traffic and node density.

The dependability and effectiveness of data transmission are reflected in the packet delivery ratio. The findings demonstrate that, in every simulation round, RL-ILEACH consistently outperforms LEACH and ILEACH in terms of PDR. Packet loss in LEACH is caused by unstable clusters and frequent CH failures. ILEACH boosts cluster stability, but when energy imbalance grows over time, packet delivery performance deteriorates. RL-ILEACH’s adaptability guarantees dependable CH selection and steady cluster formation, which reduces packet losses and boosts throughput. For mission-critical WSN applications, higher PDR in RL-ILEACH signifies improved data aggregation efficiency and less transmission overhead.

All things considered, the simulation results verify that the suggested RL-ILEACH protocol performs noticeably better than LEACH and ILEACH in terms of:

Reduced use of energy, extended period of stability, increased ratio of packet delivery and.

longer network lifespan these findings show that RL-ILEACH is a dependable, clever, and energy-efficient solution for dynamic Wireless Sensor Network situations and support the efficacy of incorporating reinforcement learning into the ILEACH protocol.

**Fig. 16:**
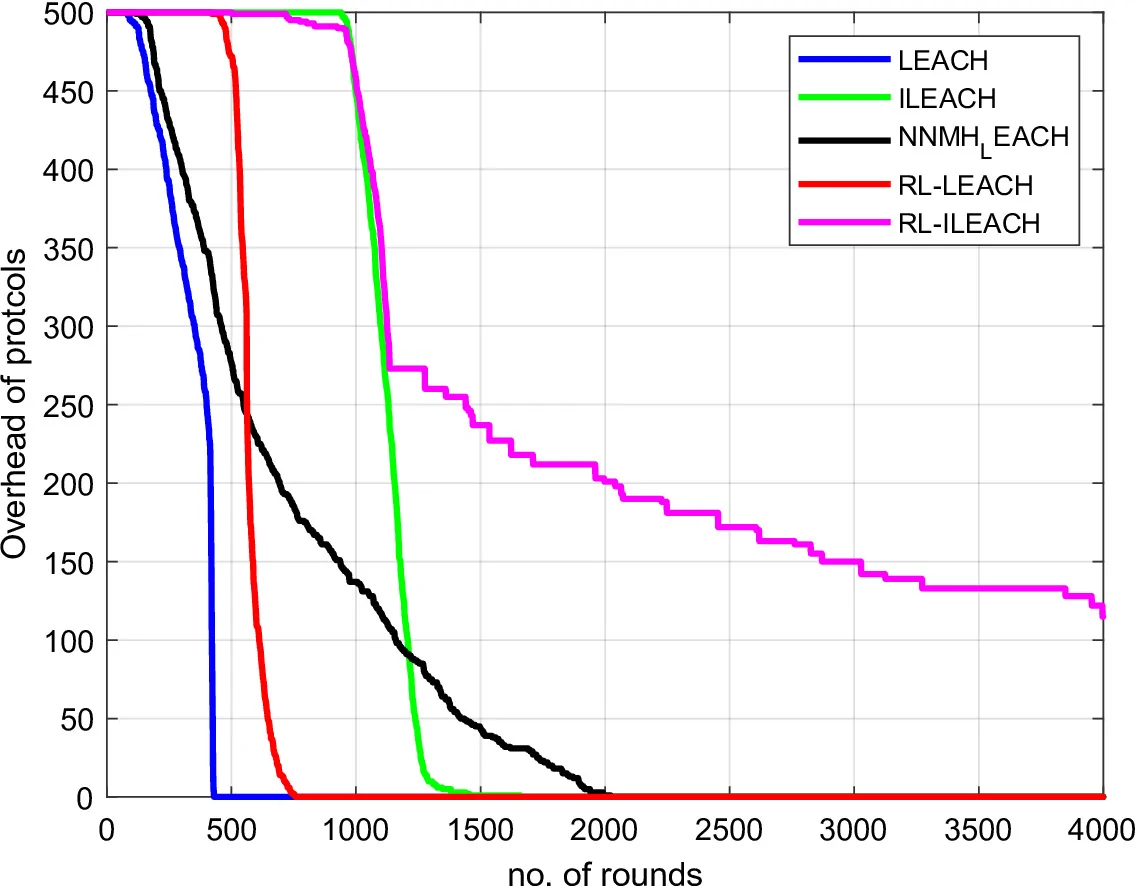
Communication overhead comparison among the evaluated protocols under the dense network scenario( 500 nodes network).

Figure 16 presents the communication overhead incurred by each protocol in the dense network scenario. This experiment evaluates the additional control messages required for clustering and routing, where lower overhead indicates greater protocol efficiency and better scalability for large-scale wireless sensor networks.

### Reward function weight justification and sensitivity analysis

The primary objective of RL-ILEACH is extending network longevity by avoiding low-energy nodes as CHs. Therefore, Residual Energy (α) is given the highest weight to heavily penalize low-energy operations. Transmission Cost (β) is weighted second to penalize long-distance, high-energy communications. Successful Data Delivery (γ) acts as a secondary regularizer to maintain data reliability.

Sensitivity Analysis: To validate the robustness of these parameters, we performed a sensitivity analysis by varying the weights across three distinct network scenarios in Table 4.

The results demonstrate that increasing α generally improves network lifetime and stability, whereas excessively large β values reduce throughput due to overly conservative transmission behavior. Increasing γ improves packet delivery ratio but may slightly increase energy consumption. The selected values achieved the best trade-off among the evaluated performance metrics.

These additions clarify the reward design process and demonstrate the robustness of the proposed approach.

**Table 4:** Sensitivity analysis for reward function’s weights.

Scenario profile	Weight configuration	Primary effect observed
Energy-Conservative (Baseline)	α = 0.5, β = 0.3, γ = 0.2	Yields the most balanced network lifetime and extends the stability period (FND).
Distance-Aggressive	α = 0.2, β = 0.6, γ = 0.2	Minimizes immediate transmission overhead but leads to localized energy holes and faster early node failure.
Reliability-Focused	α = 0.3, β = 0.2, γ = 0.5	Maximizes Packet Delivery Ratio (PDR) early on but accelerates overall network energy decay.

### Statistical discussion

To provide a more robust analysis and quantify the statistical significance of the results, a T-test and descriptive statistics were conducted. The results of the statistical analysis and T-test can be found in Tables 5, 6, 7, 8, 9, 10, 11 and 12, whch present the statistical comparison between the protocols.

**Table 5:** Descriptive statistics for allive node report.

Mean	*N*	Std. Deviation	Std. Error Mean	
leach	47.2662	3501	130.88640	2.21207
RlLeach	260.2465	3501	162.59788	2.74801
ileach	161.1808	3501	226.19529	3.82285
RliLeach	78.0588	3501	174.52819	2.94964

**Table 6:** Statistical allive node T-test report.

	Paired differences					t	df	Sig. (2-tailed)
	Mean	Std. Deviation	Std. Error Mean	95% confidence interval of the difference				
				Lower	Upper			
leach - RlLeach	-212.98029	144.56268	2.44321	-217.77054	-208.19004	-87.172	3500	0.000
ileach - RliLeach	83.12197	169.45093	2.86383	77.50701	88.73692	29.025	3500	0.000

**Table 7:** Descriptive statistical energy report.

Mean	*N*	Std. Deviation	Std. Error Mean	
leach	83.4398	467	72.50567	3.35516
RlLeach	133.5482	467	68.15153	3.15368
ileach	62.5432	2246	80.25775	1.69349
RliLeach	66.3268	2246	78.97200	1.66636

**Table 8:** Statistical energy T-Test report.

	Paired Differences					t	df	Sig. (2-tailed)
	Mean	Std. Deviation	Std. Error Mean	95% confidence interval of the difference				
				Lower	Upper			
leach - RlLeach	-50.10846	21.52221	0.000	-52.06552	-48.15139	-50.313	466	0.000
ileach - RliLeach	-3.78357	1.89611	0.04001	-3.86203	-3.70511	-94.568	2245	0.000

**Table 9:** Descriptive statistical packet delivery report.

Mean	*N*	Std. Deviation	Std. Error Mean	
leach	0.7001	3501	0.00411	0.00007
RlLeach	0.7000	3501	0.00262	0.00004
ileach	0.7000	3501	0.00304	0.00005
RliLeach	0.7000	3501	0.00321	0.00005

**Table 10:** Statistical packet delivery T-Test report.

Paired differences						t	df	Sig. (2-tailed)
	Mean	Std. Deviation	Std. Error Mean	95% confidence interval of the difference				
				Lower	Upper			
leach - RlLeach	0.00012	0.00473	0.00008	− 0.00004	0.00027	1.475	3500	0.140
ileach - RliLeach	− 0.00005	0.00457	0.00008	− 0.00020	0.00011	− 0.585	3500	0.550

**Table 11:** Descriptive statistical throughput protocols reports.

Mean	*N*	Std. Deviation	Std. Error Mean	
leach	36.9637	3501	98.74423	1.66884
RlLeach	197.5618	3501	118.42441	2.00145
ileach	118.9123	3501	166.95956	2.82173
RliLeach	56.0743	3501	127.98192	2.16298

**Table 12:** Statistical throughput T-Test reports.

Paired differences					t	df	Sig. (2-tailed)	
Mean	Std. Deviation	Std. Error Mean	95% confidence interval of the difference					
			Lower	Upper				
leach - RlLeach	-160.59811	104.46349	1.76550	-164.05963	-157.13660	-90.965	3500	0.000
ileach - RliLeach	62.83805	126.26225	2.13392	58.65420	67.02189	29.447	3500	0.000

By employing the T-test and presenting the statistical results in Tables 5, 6, 7, 8, 9, 10, 11 and 12, the study provides a quantitative assessment of the performance differences between the protocols, lending further credibility to the observed trends and confirming the superiority of RliLeach protocol in terms of lifetime, energy, throughput and packet delivery ratio. They emphasize the importance of improving network lifetime and reducing energy consumption through enhanced cluster head selection. Their findings indicate that the RlLEACH protocol significantly outperforms the original LEACH protocol and RliLeach protocol outperforms than improning iLeach protocol in terms of network lifetime and throughput, which aligns with the objectives of our research.

## Conclusion and future work

In order to improve CH selection in WSNs, this study introduced an intelligent and energy-efficient clustering protocol called RL-ILEACH, which incorporates RL into the Improved Low-Energy Adaptive Clustering Hierarchy (ILEACH) protocol. The main objective was to introduce a learning-based strategy that could adjust to changing network conditions in order to address the drawbacks of static and heuristic-based CH selection techniques. An RL agent is used in the suggested RL-ILEACH protocol to dynamically choose the best CHs depending on current network conditions, such as residual energy, node dispersion, and communication distance. RL-ILEACH attains balanced energy consumption and enhanced clustering efficiency by optimizing long-term incentives and learning from past interactions^26,27^.

Extensive MATLAB simulations were conducted under different network densities (200 and 500 sensor nodes) and compared with LEACH, ILEACH, RL-LEACH, and NNMH-LEACH. The simulation results demonstrate that RL-ILEACH consistently outperforms the existing protocols in terms of network lifetime, stability period, residual energy, throughput, packet delivery ratio (PDR), communication overhead, and the number of alive sensor nodes. Specifically, RL-ILEACH extended the network lifetime by approximately 784% compared with LEACH, 130% compared with ILEACH, and 108% compared with NNMH-LEACH. Furthermore, the proposed protocol maintained more than 120 active sensor nodes after 4000 simulation rounds, whereas all competing protocols experienced complete network failure before reaching this limit. These results confirm that the reinforcement learning-based CH selection mechanism effectively balances energy consumption, minimizes premature node failures, and significantly enhances the overall operational lifetime of the network.

Compared with existing clustering protocols, RL-ILEACH provides a practical balance between intelligent decision-making and computational efficiency. Unlike Deep Reinforcement Learning (DRL) and Multi-Agent Reinforcement Learning (MARL) approaches, which require substantial computational resources and communication overhead, the proposed tabular Q-learning framework offers adaptive behavior with minimal processing and memory requirements, making it well suited for resource-constrained WSN environments.

As future work, the proposed RL-ILEACH protocol can be extended in several directions. Future research will investigate the integration of Deep Reinforcement Learning (DQN) to Multi-Agent Reinforcement Learning (MARL) for cooperative distributed CH selection, and mobile and heterogeneous WSNs. Additional research will focus on incorporating energy harvesting techniques, security-aware clustering mechanisms, IoT-enabled edge computing architectures, and real-world hardware implementation to further improve scalability, reliability, and applicability in practical smart sensing environments.

## Data Availability

All data generated or analyzed during this study are included in this published article.
